# Histopathological Changes in the Uterus, Ovaries, and Fallopian Tubes Following Testosterone Therapy in Transgender Men: A Retrospective Cohort Study from a Tertiary Referral Center

**DOI:** 10.3390/jcm15187304

**Published:** 2026-09-20

**Authors:** Kübra Hamzaoğlu Canbolat, Hilal Atılgan Yıldırım, Nil Urgancı, Şennur İlvan, Mahmut Öncül, Koray Gök, Abdullah Tüten

**Affiliations:** 1 Department of Obstetrics and Gynecology, Cerrahpaşa Faculty of Medicine, Istanbul University-Cerrahpaşa, Istanbul 34098, Turkey; mahmutoncul@gmail.com (M.Ö.); drkorayctf@hotmail.com (K.G.); drabdtuten@gmail.com (A.T.); 2 Department of Obstetrics and Gynecology, Kartal Lütfi Kırdar City Hospital, Istanbul 34865, Turkey; drhilalatilgan@gmail.com; 3 Department of Pathology, Cerrahpaşa Faculty of Medicine, Istanbul University-Cerrahpaşa, Istanbul 34098, Turkey; nilurganci@yahoo.com (N.U.); sennurilvan@hotmail.com (Ş.İ.)

**Keywords:** transgender men, testosterone therapy, gender-affirming surgery, endometrium, ovary, histopathology, estrogen receptor, progesterone receptor, immunohistochemistry

## Abstract

**Background**: Gender-affirming testosterone therapy is an essential component of medical transition for transgender men. Despite its widespread use, the histopathological effects of testosterone exposure on the uterus, ovaries, and fallopian tubes remain incompletely characterized. This study aimed to characterize histopathological findings in the gynecologic organs of transgender men receiving testosterone therapy before gender-affirming hysterectomy with bilateral salpingo-oophorectomy. **Methods**: This retrospective cohort study included transgender men who underwent laparoscopic hysterectomy with bilateral salpingo-oophorectomy at a tertiary referral center between 2015 and 2019. Demographic characteristics, duration of testosterone therapy, operative findings, and histopathological results were reviewed. Estrogen receptor (ER) and progesterone receptor (PR) expression in uterine tissues was assessed immunohistochemically. **Results**: Fifty-two transgender men were included. The median age was 25.5 years (range, 19–46 years), the median body mass index was 24.8 kg/m^2^, and the mean duration of testosterone therapy was 16.1 ± 7.8 months. Proliferative endometrium was the predominant pattern (78.8%), followed by inactive (17.3%) and atrophic (3.8%) endometrium. Endometrial polyps were identified in 13.5% of patients. Multifollicular ovarian morphology was observed in 90.4%, and paratubal cysts in 63.5%. No premalignant or malignant lesions were detected. ER expression was positive in all endometrial samples and in 92% of myometrial specimens, while PR expression was preserved in all evaluated uterine tissues. **Conclusions**: In this cohort, proliferative endometrium and multifollicular ovarian morphology were common despite testosterone therapy, and uterine ER and PR expression remained detectable. The absence of premalignant or malignant lesions was reassuring within the observed exposure period; however, the retrospective design, modest sample size, absence of a control group, limited characterization of hormone exposure, and lack of longitudinal follow-up preclude conclusions regarding long-term oncologic safety or malignancy risk.

## 1. Introduction

Gender incongruence refers to a discrepancy between an individual’s gender identity and the sex assigned at birth. Greater social awareness and improved access to specialized healthcare have been accompanied by a steady increase in the number of transgender individuals seeking gender-affirming medical and surgical interventions worldwide. According to the World Professional Association for Transgender Health (WPATH) Standards of Care Version 8 and the Endocrine Society Clinical Practice Guideline, gender-affirming hormone therapy is a cornerstone of care for transgender individuals and is associated with improvements in gender dysphoria, psychological well-being, quality of life, and overall health outcomes [1,2].

For transgender men, testosterone therapy induces virilization through the development of secondary male sexual characteristics, cessation of menstruation, redistribution of body fat, increased muscle mass, and deepening of the voice. Although these clinical effects are well established, the biological effects of testosterone exposure on the uterus, ovaries, and fallopian tubes remain incompletely understood. Testosterone can be aromatized to estradiol, but the contribution of this pathway is tissue-dependent. Aromatase activity is well established in adipose tissue and the ovary, whereas aromatase expression is very low or undetectable in normal eutopic endometrium and may be upregulated in pathological states such as endometriosis [3]. Thus, persistent endometrial activity during testosterone therapy may reflect a combination of peripheral estrogen formation, residual ovarian steroidogenesis, local steroid metabolism, and tissue-level receptor sensitivity rather than a single mechanism.

Several mechanisms have been proposed to explain histopathological changes associated with testosterone administration. Experimental and clinical studies suggest that androgen exposure may influence endometrial proliferation, ovarian folliculogenesis, stromal remodeling, and myometrial architecture [4,5]. However, published findings remain inconsistent [6]. Some investigators have reported predominantly atrophic endometrium following testosterone therapy, whereas others have demonstrated persistent proliferative endometrium [7,8].

Similarly, ovarian morphology in transgender men may resemble that observed in polycystic ovary syndrome (PCOS), including multifollicular development and stromal changes [9,10]. Nevertheless, whether these findings represent physiological adaptation to exogenous androgen exposure or true pathological alterations remains uncertain. Concerns have also been raised regarding endometrial hyperplasia and gynecologic malignancy during testosterone therapy, although current evidence remains limited and generally reassuring [1,11].

The existing literature consists primarily of small retrospective cohorts, and comprehensive histopathological evaluations of all gynecologic organs—including immunohistochemical assessment of steroid hormone receptor expression—remain limited [12,13]. Additional evidence is therefore needed to clarify the tissue-level effects of testosterone therapy and to provide clinicians with reliable data for counseling transgender individuals considering gender-affirming surgery.

The present study aimed to evaluate histopathological changes in the uterus, ovaries, cervix, and fallopian tubes of transgender men receiving testosterone therapy before hysterectomy with bilateral salpingo-oophorectomy. In addition, we investigated estrogen and progesterone receptor expression in uterine tissues to better characterize steroid hormone responsiveness after testosterone exposure.

## 2. Materials and Methods

This retrospective cohort study was conducted at the Department of Obstetrics and Gynecology, Cerrahpaşa Faculty of Medicine, Istanbul University-Cerrahpaşa, Turkey. We retrospectively reviewed the medical records of transgender men who underwent gender-affirming laparoscopic hysterectomy with bilateral salpingo-oophorectomy between January 2015 and December 2019. The study protocol was approved by the Institutional Ethics Committee of Istanbul University-Cerrahpaşa on 7 January 2020 (Approval No. 80345809-604.01.02) and was conducted in accordance with the principles of the Declaration of Helsinki.

Eligible participants were transgender men aged ≥18 years who had received continuous testosterone therapy before surgery and who underwent hysterectomy with bilateral salpingo-oophorectomy because of gender dysphoria. Only patients with complete clinical and pathological records were included.

Patients were excluded if they had a history of abnormal uterine bleeding, a previous diagnosis of endometrial hyperplasia or malignancy, gynecologic cancer, incomplete pathology reports, or missing clinical data. Patients with a documented pre-existing diagnosis of PCOS were also excluded because baseline hyperandrogenism and PCOS-associated ovarian morphology could confound interpretation of the ovarian findings.

Demographic and clinical characteristics were extracted from hospital records. The following variables were recorded: age at surgery, body mass index (BMI), relationship status, parity, smoking status (if available), systemic comorbidities, duration of testosterone therapy before surgery, previous gynecologic history, and operative findings. All patients received masculinizing hormone therapy with intramuscular testosterone undecanoate. Testosterone dosing was individualized by an endocrinologist according to serum total testosterone concentrations, with a target range of 400–800 ng/dL. Patient-level administered doses, cumulative testosterone exposure, and contemporaneous serum testosterone and estradiol concentrations were not consistently available in the retrospective dataset and therefore could not be analyzed as dose–response variables.

The primary outcome was the histopathological evaluation of the uterus, ovaries, cervix, and fallopian tubes. Secondary outcomes included immunohistochemical expression of estrogen receptor (ER) and progesterone receptor (PR). All procedures were performed laparoscopically by experienced gynecologic surgeons according to standardized institutional protocols. After induction of general anesthesia, patients were placed in the dorsal lithotomy position with Trendelenburg tilt. Pneumoperitoneum was established using a Veress needle (Hangzhou Boer Medical Instrument Co., Ltd., Hangzhou, China), and laparoscopic hysterectomy with bilateral salpingo-oophorectomy was completed using a conventional multiport technique. Surgical specimens were immediately fixed in 10% buffered formalin and submitted for pathological examination. All specimens were evaluated by experienced gynecologic pathologists using standard pathological protocols. Endometrial morphology was classified as proliferative, secretory, inactive, or atrophic according to glandular architecture, stromal appearance, mitotic activity, and the gland-to-stroma ratio. Proliferative endometrium was characterized by proliferative-phase glandular architecture with nuclear pseudostratification and mitotic activity; secretory endometrium by glandular secretory changes and/or a corresponding stromal response; inactive endometrium by sparse glands with minimal mitotic or secretory activity; and atrophic endometrium by small inactive glands within compact or fibrotic stroma. Myometrial abnormalities, including adenomyosis, leiomyoma, calcification, and other pathological findings, were recorded. Cervical specimens were examined for inflammation, epithelial abnormalities, dysplasia, and malignancy. Ovarian tissue was evaluated for multifollicular morphology, follicular cysts, corpus luteum, endometrioma, cortical hyperplasia, and benign or malignant tumors. Fallopian tubes were examined for paratubal cysts and other pathological findings. Immunohistochemical staining for ER and PR was performed on representative uterine tissue sections. Paraffin-embedded tissues were sectioned at 2 μm and processed using the Ventana Benchmark XT automated staining platform (Roche Diagnostics, Basel, Switzerland). Monoclonal antibodies against ER (SP1 clone) and PR (1E2 clone) were used according to the manufacturer’s instructions. Immunoreactivity was evaluated by experienced pathologists on the basis of the percentage of positively stained nuclei, using routinely accepted criteria for steroid hormone receptor assessment.

Statistical analyses were performed using IBM SPSS Statistics version 22.0 (IBM Corp., Armonk, NY, USA). Continuous variables were assessed for normality using the Kolmogorov–Smirnov test. Normally distributed variables are presented as mean ± standard deviation (SD), whereas non-normally distributed variables are expressed as median (range). Categorical variables are presented as frequencies and percentages. Because the primary objective of this study was descriptive characterization of histopathological findings, analyses were limited to descriptive statistics.

## 3. Results

A total of 52 transgender men who underwent laparoscopic hysterectomy with bilateral salpingo-oophorectomy for gender dysphoria during the study period were included in the analysis. All participants had received intramuscular testosterone undecanoate before surgery, with dosing individualized by an endocrinologist to maintain serum total testosterone concentrations within a target range of 400–800 ng/dL.

The median age at surgery was 25.5 years (range, 19–46 years), and the median BMI was 24.8 kg/m^2^ (range, 21–30 kg/m^2^). The mean duration of testosterone therapy before surgery was 16.1 ± 7.8 months.

Most participants were single (88.5%), and none had a history of pregnancy. Fifteen patients (28.8%) had at least one comorbidity. The most frequently reported comorbidity was major depressive disorder (17.3%), followed by asthma (5.7%), hypothyroidism (3.8%), and migraine (1.9%).

Demographic and baseline clinical characteristics are summarized in Table 1.

Proliferative endometrium was the predominant histopathological pattern, observed in 41 of 52 patients (78.8%). Inactive endometrium was identified in 9 patients (17.3%), whereas 2 patients (3.8%) had atrophic endometrium. No secretory endometrium was identified.

Histopathological Findings are summarized in Table 2.

Endometrial polyps were present in seven patients (13.5%). No cases of endometrial hyperplasia, atypia, or endometrial carcinoma were detected. Normal myometrial architecture was observed in 34 patients (65.4%). Among myometrial abnormalities, leiomyoma was the most frequent lesion, occurring in 11 patients (21.1%), while adenomyosis was identified in one patient (1.9%). Lipogranuloma and dystrophic calcification were each detected in one patient (1.9%); the lipogranuloma was considered an incidental benign histopathological finding. No premalignant or malignant myometrial lesions were observed.

Histopathological examination of the cervix demonstrated chronic nonspecific cervicitis in 33 patients (63.4%), whereas cervical atrophy was observed in nine patients (17.3%). No cervical intraepithelial neoplasia or invasive cervical carcinoma was identified in any surgical specimen. Multifollicular ovarian morphology was the most frequent ovarian finding and was observed in 47 patients (90.4%). Additional ovarian findings included corpus luteum cysts (13.5%), simple serous cysts (5.8%), endometriomas (3.8%), benign mucinous cystadenoma (1.9%), and cortical hyperplasia (1.9%). Paratubal cysts were identified in 33 patients (63.5%) and represented the most common tubal abnormality. No borderline ovarian tumors or ovarian malignancies were identified. Immunohistochemical analysis demonstrated preserved steroid hormone receptor expression after testosterone exposure. ER expression was positive in all endometrial specimens (100%) and in 92% of myometrial samples. PR expression remained positive in all endometrial and myometrial specimens examined.

**Figure 1:**
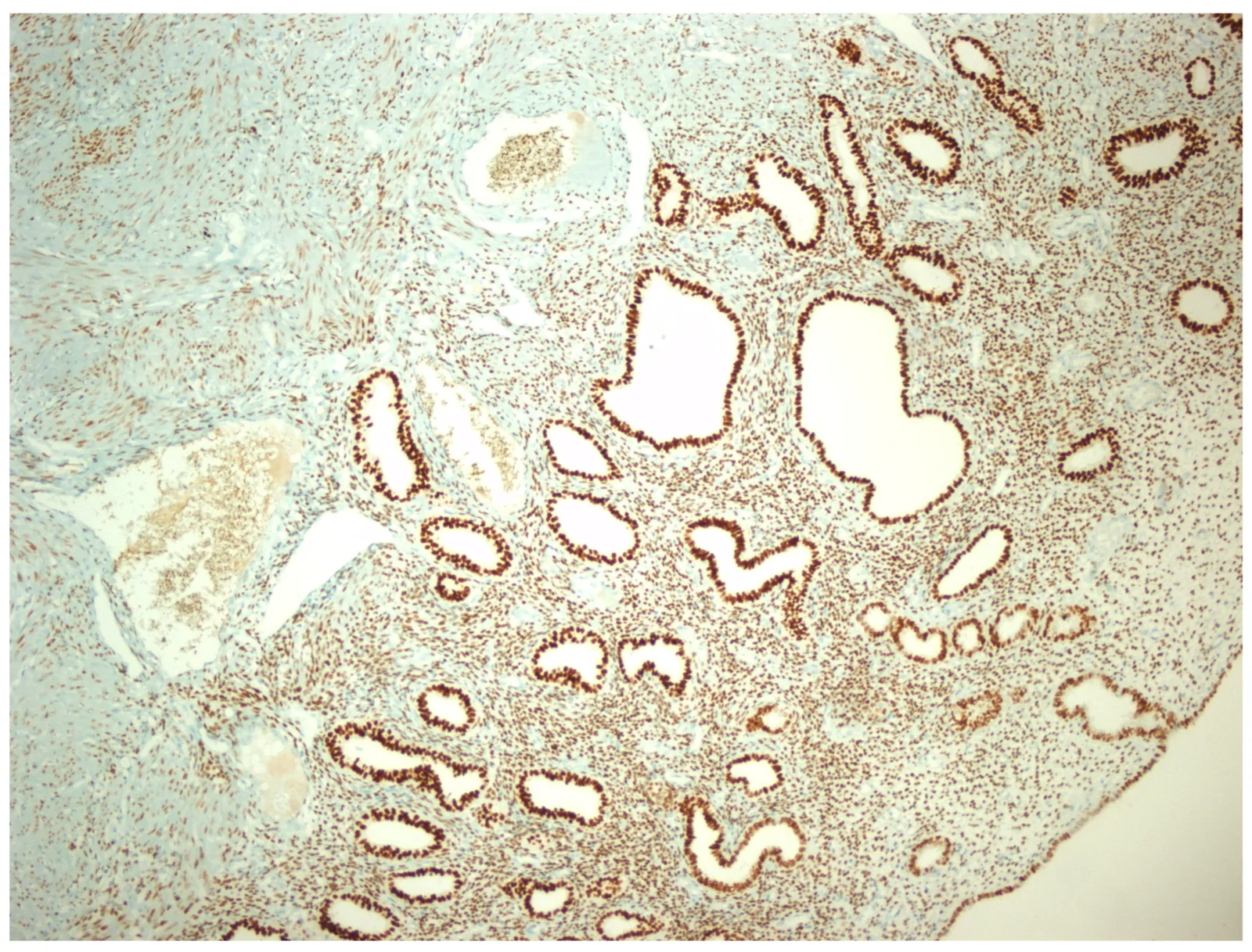
Strong estrogen receptor expression in both endometrium and myometrium magnification (magnification 100×).

**Figure 2:**
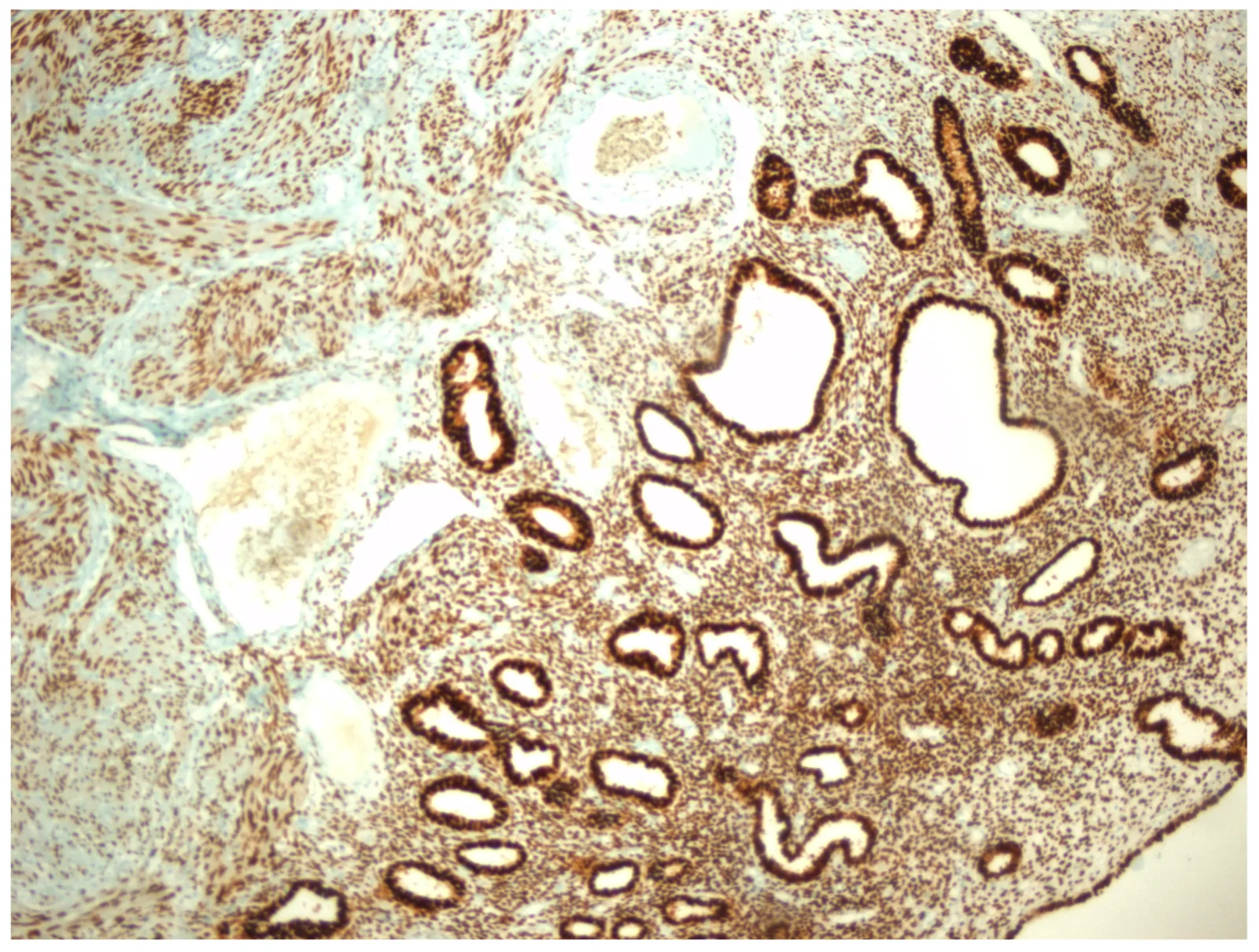
Strong progesterone receptor expression in both endometrium and myometrium magnification (magnification 100×).

Representative immunohistochemical staining patterns are shown in Figure 1 and Figure 2.

Overall, proliferative endometrium and multifollicular ovarian morphology were the predominant histopathological findings among transgender men receiving testosterone therapy. Although several benign gynecologic lesions—including leiomyomas, endometrial polyps, and paratubal cysts—were observed, no premalignant or malignant lesions were identified in any of the examined organs.

## 4. Discussion

In this retrospective cohort of 52 transgender men receiving testosterone before gender-affirming hysterectomy with bilateral salpingo-oophorectomy, proliferative endometrium and multifollicular ovarian morphology were the predominant histopathological findings. No premalignant or malignant lesions were identified after a mean testosterone exposure of approximately 16 months, and uterine ER and PR expression remained detectable. These observations add to the growing descriptive literature on the reproductive-tract effects of masculinizing hormone therapy [6,14,15]. However, given the sample size, duration of testosterone exposure, and retrospective cross-sectional nature of the histopathological assessment, the absence of malignancy should be regarded as a reassuring observation within this cohort rather than evidence of long-term oncologic safety.

The high proportion of proliferative endometrium (78.8%) is notable because published series have reported heterogeneous endometrial patterns. Grimstad et al. reported persistent endometrial activity with predominantly proliferative histology in many testosterone-treated transmasculine patients, whereas Hawkins et al. observed both proliferative and atrophic endometrium [7,8]. More recently, da Silva et al. reported atrophic endometrium in approximately two-thirds of their cohort after a longer mean duration of gender-affirming hormone therapy [16]. Differences among studies may reflect variation in age, treatment duration, testosterone formulation and route, achieved hormone concentrations, residual ovarian activity, body composition, and histopathological classification. In our cohort, formulation and route were uniform, as all patients received intramuscular testosterone undecanoate, while dosing was individualized by an endocrinologist to target serum total testosterone concentrations of 400–800 ng/dL. Nevertheless, patient-level administered doses, cumulative exposure, serial hormone concentrations, and timing relative to the last injection were not consistently available, limiting assessment of treatment-exposure effects on endometrial morphology.

Peripheral aromatization of testosterone to estradiol is one possible contributor to persistent endometrial activity, but it is unlikely to provide a complete explanation. The median BMI in our cohort was 24.8 kg/m^2^, suggesting that increased adipose aromatization alone is unlikely to account for the observed endometrial activity. Residual ovarian steroidogenesis may also be relevant: histological evidence of recent ovulatory activity has been demonstrated in a substantial proportion of amenorrheic transmasculine individuals receiving testosterone [17]. In addition, local steroid metabolism and tissue-level receptor sensitivity may influence endometrial responses. Accordingly, preserved ER expression in our specimens supports continued capacity for estrogen-responsive signaling but does not establish aromatization as the causal mechanism.

Endometrial polyps were identified in 13.5% of patients, while no endometrial hyperplasia, atypia, or carcinoma was detected. This finding is consistent with systematic and cohort-level evidence suggesting that premalignant and malignant endometrial lesions are uncommon in testosterone-treated transmasculine populations [6,15]. Nevertheless, isolated cases of endometrial carcinoma have been reported, and the available literature remains insufficient to determine whether malignancy risk changes with long-term testosterone exposure [18,19]. Persistent or recurrent uterine bleeding after the expected onset of amenorrhea should therefore prompt individualized clinical evaluation. Assessment may include review of testosterone adherence and dosing, serum testosterone measurement when clinically appropriate, pregnancy risk when relevant, medication-related causes, and structural and non-structural etiologies using the PALM-COEIN framework. Pelvic imaging and endometrial sampling should be individualized according to age, bleeding pattern, risk factors, clinical findings, and patient preferences rather than performed routinely in all patients [1,20,21].

The high prevalence of multifollicular ovarian morphology (90.4%) represents another important finding. Exogenous androgen exposure can produce ovarian morphological features that resemble PCOS, including altered follicular development and stromal changes [9,10,22]. However, multifollicular morphology during pharmacological androgen exposure is not equivalent to a diagnosis of PCOS, which requires an integrated clinical and endocrine assessment. Patients with a documented pre-existing diagnosis of PCOS were excluded from the present cohort to reduce this potential source of confounding. Nevertheless, because this was a retrospective study and systematic pre-treatment evaluation to identify previously undiagnosed PCOS was not available, an unrecognized baseline predisposition cannot be completely excluded. Accordingly, the observed ovarian morphology should be interpreted as an association in testosterone-treated patients rather than attributed exclusively to testosterone exposure.

A distinctive feature of this study was the assessment of ER and PR expression. Both receptors remained widely expressed after testosterone exposure, suggesting preservation of steroid hormone receptor expression within uterine tissues. These findings should not, however, be interpreted as evidence that estrogen and progesterone signaling dominate the hormonal milieu. Androgen receptor (AR) expression has previously been demonstrated in human ovarian and uterine tissues after long-term androgen exposure [23], and a recent study evaluating ER, PR, and AR in testosterone-treated transgender men reported limited endometrial AR expression alongside predominantly atrophic histology [16]. Because AR was not assessed in our study, we cannot characterize the relative contribution of androgen versus estrogen/progesterone receptor pathways. Future studies incorporating all three steroid receptors together with circulating hormone levels and tissue-level signaling markers would provide a more complete mechanistic framework.

Taken together, our findings indicate that the gynecologic tissues of testosterone-treated transgender men can remain biologically active and exhibit heterogeneous responses rather than uniform atrophy. The absence of premalignant or malignant pathology in this cohort is reassuring within the observed exposure period but should not be interpreted as evidence that such lesions cannot occur or that testosterone has a protective oncologic effect. Current evidence does not support prophylactic hysterectomy solely to prevent endometrial cancer in asymptomatic individuals receiving testosterone; clinical management should instead be individualized and symptom-driven [1,6].

The principal strengths of this study include standardized pathological evaluation of all surgical specimens, immunohistochemical assessment of ER and PR expression, and inclusion of consecutive patients treated at a tertiary referral center using a uniform surgical approach. The study also evaluated the uterus, cervix, ovaries, and fallopian tubes within the same cohort, allowing a broad descriptive assessment of reproductive-tract histopathology.

Several limitations should be acknowledged. The retrospective design limits causal inference, and the modest sample size limits precision for rare outcomes, including premalignant and malignant lesions. The absence of a cisgender control group is particularly important because it prevents determination of whether the observed prevalence of proliferative endometrium, leiomyomas, polyps, multifollicular ovaries, or paratubal cysts differs from background prevalence in an age-comparable population; consequently, relative risks attributable to testosterone cannot be estimated. All patients received intramuscular testosterone undecanoate, with dosing individualized by an endocrinologist to target serum total testosterone concentrations of 400–800 ng/dL; however, patient-level administered doses, cumulative exposure, serial serum testosterone and estradiol concentrations, adherence data, and systematic longitudinal postoperative follow-up were unavailable or incompletely documented. Patients with a documented pre-existing diagnosis of PCOS were excluded; however, systematic pre-treatment evaluation to identify previously undiagnosed PCOS was not available. These limitations restrict assessment of dose–response relationships, potential modifiers of histopathological response, and long-term oncologic risk. Prospective multicenter studies with appropriate comparison groups, standardized hormone exposure data, longitudinal endocrine measurements, and predefined pathological criteria are warranted.

Despite these limitations, the present findings contribute clinically relevant histopathological and immunohistochemical data to an area in which evidence remains limited. They emphasize that amenorrhea during testosterone therapy does not necessarily imply a uniformly inactive endometrium and that multifollicular ovarian morphology should be interpreted cautiously in the context of exogenous androgen exposure. These observations may assist clinicians and pathologists in counseling patients and interpreting reproductive-tract findings while avoiding overstatement of the currently available safety data.

## 5. Conclusions

In conclusion, proliferative endometrium and multifollicular ovarian morphology were common in this cohort of transgender men receiving testosterone therapy, while ER and PR expression remained detectable in uterine tissues. No premalignant or malignant lesions were identified in this cohort. Although reassuring within the observed exposure period, this finding should not be interpreted as evidence of long-term oncologic safety or reduced malignancy risk. The retrospective design, modest sample size, absence of a control group, limited characterization of testosterone exposure, and lack of longitudinal follow-up preclude such conclusions. Prospective controlled studies integrating histopathological, endocrine, and molecular data are needed to characterize the long-term effects of masculinizing hormone therapy on reproductive tissues.

## Figures and Tables

**Table 1:** Demographic and Clinical Characteristics.

	Min–Max (Median) or n (%)
Age, years	19–46 (25.5)
Body mass index, kg/m^2^	21–30 (24.8)
**Relationship status**	
Single	46 (88.5%)
In a relationship	6 (11.5%)
**Comorbidities**	
Asthma	3 (5.7%)
Hypothyroidism	2 (3.8%)
Major depressive disorder	9 (17.3%)
Migraine	1 (1.9%)

**Table 2:** Histopathological Findings.

	n (%)
**Endometrium**	
Atrophic endometrium	2 (3.8%)
Inactive endometrium	9 (17.3%)
Proliferative endometrium	41 (78.8%)
Secretory endometrium	0
Endometrial polyp	7 (13.5%)
Adenomyosis	1 (1.9%)
Leiomyoma	11 (21.1%)
Lipogranuloma	1 (1.9%)
Myometrial calcification	1 (1.9%)
**Ovaries**	
Multifollicular morphology	47 (90.4%)
Corpus luteum cyst	7 (13.5%)
Endometrioma	2 (3.8%)
Simple serous cyst	3 (5.8%)
Benign mucinous cystadenoma	1 (1.9%)
Cortical hyperplasia	1 (1.9%)
**Fallopian tubes**	
Paratubal cyst	33 (63.5%)
**Cervix**	
Atrophy	9 (17.3%)
Chronic nonspecific cervicitis	33 (63.4%)

## Data Availability

The datasets generated and/or analyzed during the current study are available from the corresponding author upon reasonable request.

## Abbreviations

BMI	Body Mass Index
SPSS	Statistical Package for the Social Sciences
SD	Standard Deviation
